# Is health, growth and development impaired in children who are Hepatitis B-exposed but uninfected?

**DOI:** 10.1371/journal.pgph.0004984

**Published:** 2025-08-19

**Authors:** Sheila F. Lumley, Elaine Parker, Andrew J. Prendergast, Philippa C. Matthews

**Affiliations:** 1 Nuffield Department of Medicine, University of Oxford, Oxford, United Kingdom; 2 Department of Infectious Diseases and Microbiology, Oxford University Hospitals, Oxford, United Kingdom; 3 Blizard Institute, Queen Mary University of London, London, United Kingdom; 4 Zvitambo Institute for Maternal and Child Health Research, Harare, Zimbabwe; 5 The Francis Crick Institute, London, United Kingdom; 6 Division of Infection and Immunity, University College London, London, United Kingdom; 7 Department of Infectious Diseases, University College London Hospital, London, United Kingdom; University of Michigan, UNITED STATES OF AMERICA

## Abstract

An estimated 254 million people are living with chronic hepatitis B virus (HBV) infection worldwide. Many infants are born to mothers with HBV but do not themselves acquire the infection. It is unclear whether this exposure to HBV in early life - without the development of active infection - may be associated with adverse outcomes. We propose the term “HBV-exposed uninfected (HBEU)”, drawing parallels with the HIV field which recognises that children who are HIV-exposed but uninfected face an increased risk of adverse health outcomes. This paper explores the potential health consequences for children HBEU. We summarise existing evidence reporting on children HBEU, and also review existing knowledge from the HIV field that could inform insights. We hypothesise that children HBEU may be at increased risk of preterm birth, and/or impaired growth and neurodevelopmental delay, but comprehensive, longitudinal studies are currently lacking to support this. We propose a conceptual framework to hypothesise how exposure to HBV could potentially lead to adverse growth and neurodevelopment through both HBV-specific and universal pathways, and review the available evidence and research gaps. Data are needed to establish whether short- and long-term sequelae exist for children HBEU, and to inform evidence-based interventions to mitigate against detrimental outcomes. Establishing a comprehensive understanding of the long-term trajectory of health and well-being among children HBEU throughout childhood into adolescence will require longitudinal observational studies with appropriate control groups to characterise outcomes, identify risk factors and explore underlying mechanistic pathways.

## 1. Background

Worldwide, an estimated 254 million people are living with chronic hepatitis B virus (HBV) infection (PLWHB), and an estimated 4.5 million women living with HBV infection give birth each year [1–3]. In this article, we focus on the population of infants who are exposed to HBV *in utero* or peri-partum but who do not acquire the infection. For the first time, we propose the term *“HBV-exposed uninfected (HBEU)”* to describe this group which has not previously been formally defined. We build on concepts from the HIV field, in which “*children who are HIV-exposed but uninfected (CHEU)”* are at increased risk of impaired growth and neurodevelopment [4,5], and higher morbidity and mortality [6–8] compared to HIV-unexposed children. These differences in growth and development can be attributed both to HIV-specific pathways (which involve biological influence of the virus, immune activation and antiretroviral drugs) and universal pathways (through amplification or enrichment of common risk factors for poor growth and development) [5].

HBV may likewise have adverse impacts on child growth and development through biological and universal pathways. Longstanding neglect of clinical and public health infrastructure to enhance prevention, diagnosis, risk-stratification and treatment of HBV has contributed to the vulnerability of high-endemicity populations in resource-limited settings [9]. HBV prevalence is highest in populations in WHO African, South East Asian and Western Pacific regions, but has been neglected as a public health threat, with specific evidence-gaps for Africa (which now represents the region of highest new incidence due to vertical transmission) [1,9,10]. In lower prevalence settings, a significant proportion of HBV in women of reproductive age may therefore be accounted for by migrant populations [11].

Whilst strengthening the public health response to tackling paediatric HBV – now combined with HIV and syphilis in a triple elimination agenda [12] – there is an opportunity to simultaneously address the needs of children HBEU. While some published data suggest health consequences of early-life exposure to HBV (**Table 1**), long-term consequences for children HBEU have not been studied. Here, we propose and explore the hypothesis that there may be health consequences for children HBEU, to stimulate dialogue in clinical, research and public health communities, and to propose a framework for further research.

**Table 1:** Selected studies identified by a narrative review providing evidence for outcomes in infants and children HBEU. For areas where meta-analyses were available, only the meta-analyses have been reported here and other research types were excluded (e.g., pregnancy and birth outcomes). For areas where meta-analyses were not available, we present all primary literature (e.g., infant growth, vaccine response and neonatal immunity).

Reference	Study type	Setting	Findings (red = increased risk)
**Pregnancy**			
Afraie 2023 [33]	Meta-analysis	China, Sweden, Hong Kong, USA, Germany, Thailand, Myanmar- Thailand border	**Pre-eclampsia:** RR: 1.10 (95% CI 1.04, 1.16) **GDM**: 1.16 (1.13, 1.18) **Abortion**: 0.97 (0.71, 1.33) **Eclampsia**: 1.48 (0.95, 2.29)
Jiang 2020 [100]	Meta-analysis	China, Sweden	**Intrahepatic cholestasis of pregnancy**: OR 1.68 (1.43,1.97)
Birth*			
Afraie 2023 [33]	Meta-analysis	China, Sweden, Hong Kong, USA, Germany, Thailand, Myanmar- Thailand border	**PTB**: RR 1.17 (95% CI 1.14, 1.20) **Neonatal death**: RR 0.83 (0.67, 1.03)
Huang 2023 [148]	Meta-analysis	Myanmar-Thailand border, China, Sweden, USA, Korea	**Congenital abnormalities**: pooled cRR of 1.15 (95% CI: 0.92, 1.45)
Ma 2018 [32]	Meta-analysis	Greece, China, Sweden, USA, Hong Kong, Thailand, Israel, Singapore, Iran	HBsAg + /HBeAg- women vs uninfected: **Preterm labour:** pooled OR 1.28; (95% CI, 1.02, 1.59) **PTB**: pooled OR 1.16 (1.04, 1.29) HBsAg + /HBeAg+ women vs uninfected: **PTB**: pooled RR 1.21; 95% CI 1.10, 1.32; *P * < 0.001
Cui 2017 [31]	Meta-analysis	Hong Kong, Greece, Israel, USA, Germany, Singapore, Thailand, China	**PTB**: pooled RR 1.26 (95% CI 1.19, 1.33)
**Infant growth**			
Zhang 2017 [39]	Cohort study	China	**Head circumference** at 18 months: 3mm greater among children in non-HBV exposed group (47.3 ± 1.3) cm than children HBEU (47.0 ± 2.0) cm (*P = *0.038). No difference in height, weight, increases in height/weight/head circumference each month, weight/height/head circumference for age Z scores, proportion of growth retardation and low weight.
**HBV vaccine response**			
Jiang 2023 [46]	Cohort study	China	2163 infants HBEU, with/without passively acquired HBeAg. Neonatal HBeAg positivity correlated with transiently lower anti-HBs at 7-12m of age, no difference by 24m of age.
Huang 2019 [47]	Cohort study	China	265 infants born to mothers HBsAg positive, HBeAg pos/neg. Cord blood HBeAg status did not correlate with lower anti-HBs at 7-12m of age.
Wang 2017 [41]	Cohort study	China	328 mother-infant pairs, mother living with HBV. In 318 infants HBEU, non-response to HBV vaccine (anti-HBs < 10mIU/ml) was associated with transient post-natal HBsAg positivity (all infants tested HBsAg negative by 7 months)
Koumbi 2010 [48]	Cohort study	Greece	46 mother-children HBEU infant pairs, mothers HBsAg + /eAg- vs. HBsAg neg. All neonates responded to vaccination and HBEU. 50% had viral antigen induced IFNg suggesting prior exposure to viral antigens, however this did not impair T cell response to vaccination.
Badur 1994 [42]	Cohort study	Turkey	14 infants HBEU. 22% did not produce anti-HBs 1 month after 3rd dose of HBV vaccine, all of whom had detectable HBV DNA in fetal PBMC at birth.
**Neonatal immunity**			
Hong 2015 [49]	Cohort study	Singapore	HBV exposure *in utero* associated with a state of trained immunity, characterised by innate immune cell maturation and Th1 development. NB. HBV infection status not reported.
Shrivastava 2013 [149]	Cohort study	India	22 newborns born to mothers living with HBV . Newborns born HBsAg-ve (n = 12) maintain CD8 + T cell function (IFNg production and CD107A expression) v.s. HBsAg +ve (n = 10) newborns. NB. All subsequently given HBV-BD vaccine, infection status at 12 months not reported.

Acronyms: BD = birth dose, HBV = hepatitis B virus, HBEU = hepatitis B exposed uninfected, PBMC = Peripheral Blood Mononuclear Cells, SGA = small for gestational age, LBW = low birth weight, PTB = preterm birth, GDM = gestational diabetes mellitus, ICP = intrahepatic cholestasis of pregnancy, IUGR = intrauterine growth restriction, LGA = large for gestational age, PROM = preterm rupture of membranes, RCT = randomised controlled trial

* NB. These studies reported outcomes in mothers living with HBV, regardless of infant infection status.

## 2. Search strategy

The aim of the paper is to develop hypotheses about outcomes in children HBEU, assimilating a theoretical body of data which can be applied to inform the development of new evidence. As there are insufficient data for a systematic review, we adopted a narrative review approach. We undertook our search of the PubMed database between March 2023 - April 2024. We used search terms for “HBV”, “neonate/infant/child”, “growth/neuro-development” and “exposure/uninfected” using MeSH terms which were supplemented with relevant free text terms (truncated where appropriate to capture maximum resources). We narrowed the search to incorporate areas of interest, (including specific birth outcomes, microbiome, prevention of mother to child transmission (‘PMTCT’), breastfeeding). The search result was limited to articles that were written in English with a priority to include publications from the last 10 years. For areas where meta-analyses were available, we focus on reporting the meta-analysis data and other research types were excluded (e.g., pregnancy and birth outcomes). For areas where meta-analyses were not available, we present the primary literature (e.g., infant growth, vaccine response and neonatal immunity). The database searches were complemented with manual review of the reference lists of relevant articles.

## 3. Children who are HIV- vs. HBV-exposed uninfected

**Fig 1:**
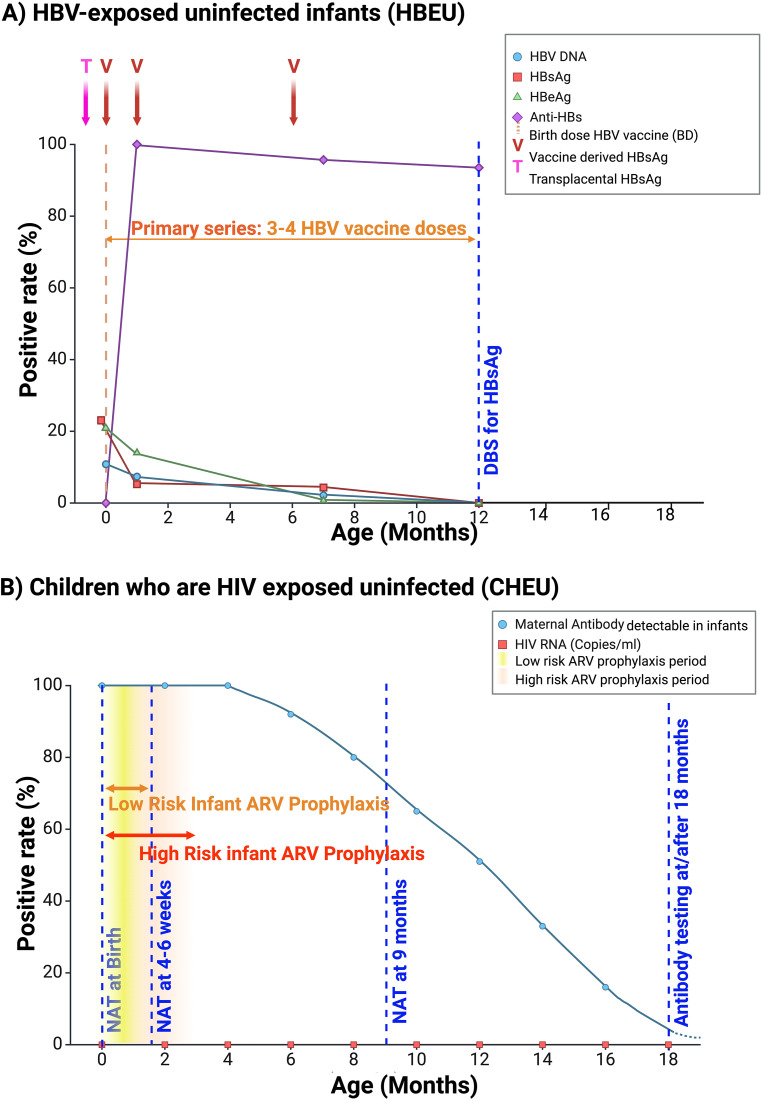
Comparison between infant viral markers, prophylaxis, treatment and testing strategies for prevention of vertical transmission of HIV and HBV [25,152,160]. (A) HBV exposed uninfected infants (HBEU). Transient HBsAg positivity is seen due to Transplacental transfer of HBsAg (**T**) and Vaccine derived HBsAg (**V**), highlighting the need for delayed testing and confirmation of infant HBV infection status at 12 months of age. Data adapted from a single study [25] of 139 HBEU infants who received combined HBV immunoprophylaxis with HBIG and HepB-BD vaccine followed by two further doses of HBV vaccine at 1 and 6 months of age (indicated on graph). WHO guidelines recommends universal HBV immunisation, including HepB-BD, to be given during infancy with a minimum of three doses, however a total of four doses is recommended. The timing of the fourth dose varies between country schedules, however recommendation is that each dose is separated by at least 4 weeks [25,152,160]. (B) Children who are HIV exposed but uninfected (CHEU). Transient maternal HIV-1 antibodies can take 18 months or longer to be cleared, therefore testing for chronic HIV infection in infants prior to 18 months of age using antibody tests is inaccurate. WHO advice serological antibody testing at 18 months or 3 months following cessation of breastfeeding [152]. HIV RNA levels remain undetectable in CHEU throughout the first 18 months of life. HIV antibody has been detected after 18 months of age. See WHO guideline for details of low/high risk infant ARV prophylaxis [152]. NAT - Nucleic acid test, DBS - Dried blood spot, ARV - Antiretroviral medication. Created in BioRender. Parker, E. (2024) https://BioRender.com/i19y222.

The hypotheses presented here mirror concepts in the HIV field. There are sparse data available for HBEU and many useful parallels can be drawn between HBV and HIV; however there are also key differences which influence how children HBEU may be defined, how problems may manifest, and how children HBEU should be identified (**Table 2** and **Fig 1**). Key differences include the ongoing risk of postpartum breast milk transmission in HIV compared to HBV, testing time-points, and the evolving prevention landscape for each infection.

**Table 2:** Comparison of HBV and HIV transmission, testing, treatment and prophylaxis. Testing, treatment, prophylaxis and elimination targets based on WHO guidelines [12,150–152].

	HIV	HBV
**Natural history (without intervention)**	Percentage vertical transmission without intervention 15–45% Timing of vertical transmission [153]: - 10–20% intrauterine; - 35–45% intrapartum; - 35% postpartum (via breastfeeding).	Percentage vertical transmission without intervention: HBeAg+ 70–90%, HBeAg- 10–40% [13–16] Timing of vertical transmission: - 5–10-% intrauterine [154,155]; - 90–95% intrapartum [156]; Potential also for horizontal transmission during early childhood (1–5 years).
**Infant testing**	Longitudinal molecular testing required due to ongoing transmission risk via breastfeeding and passive transfer of maternal antibody, e.g., birth, 4–6 weeks, 9 months, 18 months and end of breastfeeding risk period [157].	Testing with HBsAg recommended at 12 months [17]. Passive acquisition of viral DNA and antigens, in addition to vaccine-derived HBsAg in uninfected infants complicates earlier testing.
**Treatment and prophylaxis during pregnancy + /- breastfeeding**	Lifelong ART for all pregnant and breastfeeding women living with HIV.	Tenofovir during pregnancy and early postpartum if HBV VL ≥ 200,000 IU/mL or HBeAg positive. If VL or HBeAg testing not available, prophylaxis can be offered to all who test HBsAg positive.
**Infant prophylaxis**	ART duration dependent on risk status (4–6 weeks for low risk, 12 weeks for high risk).	HBV-BD vaccination followed by accelerated primary HBV vaccination course. HBIg if VL ≥ 200,000 IU/mL or HBeAg positive.
**Elimination targets** [12]	Zero new infections among infants and young children and achievement of the ‘95-95-95’ targets. **EMTCT impact targets:** Population case rate of new paediatric HIV infections due to MTCT of <50 cases per 100,000 live births MTCT rate of HIV of <2% in non-breastfeeding populations or <5% in breastfeeding populations.	95% reduction in incidence of chronic HBV infections. **EMTCT impact targets:** <0.1% prevalence HBsAg in children 0–4 years old. Additional target <2% MTCT rate (for countries using targeted timely HBV-BD vaccine).
**Global coverage of VT prevention programmes**	84% global coverage of ART in 2023.	45% coverage globally of HBV-BD vaccination in 2023 [158]. HBIg coverage 13% globally.
**Number of children estimated to be born exposed uninfected**	16.1 million global population (2023 [159]), 14.6 million of whom are in WHO African region.	Not previously estimated.

Acronyms: HBV-BD = hepatitis B birth dose vaccine, HBsAg = hepatitis B surface antigen, HBeAg = hepatitis B e-antigen, VL = viral load, HBIg = Hepatitis B immunoglobulin, ART = antiretroviral therapy, MTCT = mother to child transmission, EMTCT = elimination of MTCT.

## 4. Perinatal HBV exposure in children

Vertical transmission (also referred to as ‘mother-to-child transmission’ (MTCT), typically occurring peri-partum but also potentially *in utero*) is the commonest route of HBV infection worldwide. Without intervention, the rate of vertical transmission from mothers testing HBeAg-positive is between 70–90%, and if HBeAg-negative 10–40% [13–16]. Vertical transmission can be prevented with interventions prenatally (antenatal HBsAg screening ± antiviral prophylaxis), perinatally (hepatitis B birth dose vaccine (HepB-BD) ± hepatitis B immunoglobulin, HBIg) and postnatally (3–4 follow-up vaccine doses) [17]. This vaccine schedule leads to immunological protection in ~95% of children, although variability in development of anti-HBs antibodies post-vaccination has been observed, and is lower in infants who are HIV-infected [18]. In 2000, HepB-BD coverage within 24 hours of birth was 5% globally, rising to 45% in 2023. This is set to improve further, as in 2023 Gavi (the Vaccine Alliance) recommitted to supporting the introduction of HepB-BD vaccine, in addition to hexavalent infant vaccination which delivers HBV vaccine alongside other routine childhood immunisations in the first 2–6 months after birth [19]. As HBsAg testing, antenatal tenofovir prophylaxis, and BD vaccine coverage expands, the majority of children will be protected against chronic HBV infection, and therefore among all children perinatally exposed to HBsAg, the proportion of those born HBEU will increase. The absolute number of children HBEU will also depend on the number of children being born to women with CHB, influenced by the number of women of childbearing age with CHB worldwide [20] and changes in fertility rate. The global response to HBV elimination is off track, and in many populations (despite HBV being incorporated into routine vaccine programmes since the mid-1990’s), population prevalence of HBV is still moderate or high [21].

## 5. Serological profiles in diagnosis of children HBEU

To define a child as HBEU, it is critical to rule out HBV infection, which is typically based on detection of the viral envelope protein, hepatitis B surface antigen (HBsAg). Excess HBsAg is secreted as non-infectious particles, which may have a role in immune modulation [22]. Hepatitis B e-antigen (HBeAg) is a viral accessory protein secreted from infected cells, is associated with high viraemia, and has an immunomodulatory function [22]. The fetus may be exposed to very high loads of both HBsAg and HBeAg *in utero*; these antigens can be passively transmitted transplacentally and intrapartum, and can therefore be detected in uninfected infants for up 12 months postpartum [23–25]. Furthermore, HBsAg may also be detectable in blood up to 21 days days following a dose of HBV vaccination [26,27]. It is therefore difficult to use HBsAg as a test of HBV infection in early life. Most guidelines recommend HBsAg testing at 12 months of age [17,28,29], which maximises the chance of detecting all-cause infant HBV infection during the first year of life, and reduces the risk of misinterpreting placentally-transferred or vaccine-derived HBsAg as active infection [30]. Timing of HBV exposure, infection and diagnostic tests differ from HIV (**Table 2**; **Fig 1**) with definition of HBEU ultimately depending on confirmed perinatal exposure but persistent absence of HBV infection in the infant (**Table 3**).

**Table 3:** Classification of perinatal HBV exposure and infection.

		Maternal HBsAg status during pregnancy	
		Positive	Negative
**Infant HBsAg status at 1 year**	Positive	HBV infected infant – likely vertical transmission, also possible early life horizontal transmission	HBV infected infant - horizontal transmission
	Negative	HBV exposed uninfected (children HBEU)	Non-exposed, non-infected

## 6. Adverse impact of maternal HBV on birth and childhood outcomes

Several meta-analyses, including data from China, Hong Kong, Singapore, USA, Sweden, Germany, Greece, Thailand-Myanmar border, Israel and Iran, have documented an association between maternal HBV infection and increased risk of preterm birth (PTB) [31–33], which may be specifically influenced by maternal HBeAg status [32,34,35]. Due to the mixed evidence base, we only report the meta-analyses here. However, evidence is not consistent on the association between HBV exposure during pregnancy and PTB, and more data are needed to understand specific risk factors and causality. Irrespective of cause, small vulnerable newborns, who are either low birthweight (LBW), preterm (born alive before 37 weeks’ gestation) and/or small for gestational age (SGA; born with birthweight below the 10th centile for gestation) [36] are at increased risk of both short- and long-term health consequences including neonatal mortality and morbidity, delayed growth, neurodevelopmental delay, and non-communicable diseases later in life [37,38].

Minimal data on growth and neurodevelopment in children HBEU have been published. A population cohort study in China showed a smaller head circumference (47.3 + /- 1.3 cm vs. 47.0 + /- 2.0 cm, p = 0.038) but no other difference in growth parameters at 18 months of age in children born to mothers living with HBV vs. women uninfected with HBV (abstract only available) [39]. Vertical transmission of HBV at 18 months was 1.0% (1/97); child development was not assessed.

There are several caveats in interpreting current data. First, studies of birth outcomes are inconsistent in reporting infant HBV testing, therefore it is not possible to distinguish the impact of perinatal HBV exposure from infection. Second, a notable data gap exists for the WHO African region, which has a high HBV prevalence and incidence (accounting for ⅔ of new incident infections worldwide) [1], but is not represented in any meta-analyses of birth and pregnancy outcomes. Due to the diverse factors that influence outcomes, the effects of HBV exposure and infection may be different between population settings, and more complete global data are required.

## 7. Potential pathways for negative health outcomes in children HBEU

### 7.1. Specific immune responses to HBV

The impact of HBV on the neonatal immune system has largely been studied in the context of vertical transmission, with less attention given to children HBEU. The ultimate outcomes of HBV exposure and either infection or immunity are a complex interplay between natural and vaccine induced immune responses. In the case of active immunisation, the infant must mount a timely immune response to vaccine-antigen. HepB-BD vaccine plus follow-up doses prevent vertical transmission in ~95% infants, demonstrating that most HBV-exposed infants are able to mount a neutralising antibody response.

It is possible that immune response to vaccine may be altered by high exposure to viral antigens and/or DNA *in utero,* contributing to the ~ 5% of vaccinated infants HBEU who have anti-HBs titres <10 IU/ml after receiving ≥3 vaccine doses [40]. Transplacental HBsAg transfer has been associated with non-response to HBV vaccination [41], as has the transplacental passage of HBV DNA and presence of HBV DNA in neonatal PBMCs [42]. However, although it has been proposed that neonatal exposure to maternal HBeAg contributes to immune tolerance [43–45], exposure to maternal HBeAg in children HBEU does not appear to have a long-term impact on anti-HBs titres post-vaccination [46–48].

### 7.2. HBV-specific effects on immune response to other pathogens

HBV exposure might also influence the neonatal or infant immune response to other pathogens, either with beneficial or detrimental effects on morbidity and mortality. Analysis of cord blood demonstrated increased innate immune cell maturation and Th1 development in HBV-exposed compared to HBV-unexposed neonates, with increased activation of CD123^+^ plasmacytoid dendritic cells and natural killer cells, mature cord blood monocytes, low IL-10 and high IL-12p40 and IFNα2 [49]. This picture is consistent with a ‘trained immunity’ hypothesis, in which exposure to bacterial or viral infections and live vaccines can protect infants against unrelated pathogens by boosting functional innate immunity [50,51]. Such a profile in children HBEU might confer advantages upon exposure to unrelated pathogens. This is analogous to murine studies of CMV, in which exposure augments responses to other unrelated pathogens such as Listeria [52].

### 7.3. Maternal immune/inflammation mediated effects

Oxygen delivery, exposure to maternal hormones, immune activation/inflammation and toxins, play a critical role in shaping fetal development. ‘Maternal immune activation’ (MIA), as a result of infections and inflammation in general, can impact fetal growth and development through an increase in pro-inflammatory cytokines (e.g., IL-6 and activation of Th17 cells) which may potentially affect child growth and development, including cognitive and physical impairments that persist beyond infancy [53]. Maternal stress or infection can also lead to increases in pro-inflammatory cytokines leading to endocrine disruption (e.g., via the hypothalamic-pituitary-adrenal axis). This can lead to neurodevelopmental reprogramming via epigenetic changes such as DNA methylation of promoter regions (e.g., the glucocorticoid receptor promoter, leading to altered glucocorticoid receptor expression in the hippocampus [54]) and histone methylation (e.g., influencing differentiation of neural precursor cells) and therefore alter fetal growth and development [55,56]. HBV infection has the potential to modify these components of the maternal milieu, thereby impacting childhood outcomes. However, neither the impact of MIA nor neurodevelopmental reprogramming has been characterised in children HBEU.

Direct viral effects may also arise through placental infection and inflammation. Placental HBV infection is recognised [57], which can cause trophoblast apoptosis [58] allowing HBsAg, HBeAg and DNA to cross the placenta [41,59]. Other maternal infections during pregnancy have been shown to affect the developing fetal immune system (independently of the vertical transmission of pathogens) through sensitisation to pathogen antigens after transplacental transfer, inflammatory responses in placental syncytiotrophoblasts, and reduced transmission of maternal antibodies [60]. It is therefore feasible that placental HBV infection could impact the structure and morphology of the placenta and influence outcomes in children HBEU.

A rise in the liver enzyme alanine aminotransferase (ALT) during pregnancy may be evidence of maternal immune activation in HBV, due to a cytotoxic T-cell response against infected hepatocytes. Typically, such a ‘flare’ would be associated with reduced viraemia, and the potential for clearance of HBsAg [61]; however, a rise in ALT can also signify an increase in viraemia (e.g., reflecting transient immunosuppression related to pregnancy [61]), and/or could be mediated by other liver disease during pregnancy (e.g., obstetric cholestasis, preeclampsia and HELLP syndrome [62]). Such ALT flares are typically mild or self-limiting during pregnancy and postpartum [63] and some data suggest that ALT flares in HBV in the third trimester are not linked to adverse outcomes [64]. However, other studies have shown a link between raised ALT in pregnancy and the development of gestational diabetes or PTB [65]. Links between high ALT levels in HBV and cytokines such as IFN gamma, IL-2 and IL-17 producing CD4 + cells, suggesting potential mediation of a systemic inflammatory response [66], but the clinical significance on the fetus has not been determined.

### 7.4. Microbiome effects

Maternal gut microbial dysbiosis in HIV infection can be associated with adverse birth outcomes including LBW [55,67–69], and there is also evidence that HIV exposure influences the gut microbiome of HIV-uninfected infants resulting in over-maturation and over-diversification [70]. Microbial dysbiosis has also been reported in chronic HBV infection [71–77], with evidence of increased microbial translocation (e.g., evidenced by systemic LPS and sCD14), markers of enterocyte death (I-FABP) and markers of systemic inflammation [73]. On these grounds, disruption to the maternal microbiome could be a pathway to adverse fetal or infant outcomes. Influences of the maternal gut and vaginal microbiome on fetal growth and development include delivery of metabolites essential for fetal growth, and vertical transmission of the maternal microbiota peripartum [78]. It is also thought that adaptations to the maternal gut microbiome are necessary for development of the fetal gut-brain axis both pre- and post-natally, with studies citing impacts on fetal brain neurogenesis, impact on the developing fetal immune system (specifically fetal regulatory T cells), and deficiencies in short chain fatty acids impacting fetal enteric nervous system development [79]. Furthermore, it has been hypothesised that microbial dysbiosis may impact infants’ ability to clear HBV. In a mouse model it was demonstrated that 12-week-old mice with a stable microbiome cleared HBsAg within 6 weeks following injection with HBV; however, those who had not fully developed their gut microbiome by 6 weeks did not clear HBsAg by 26 weeks. This study posited an immunomodulatory role of the gut microbiome, suggesting an impact on HBV clearance [74].

Environmental enteric dysfunction (EED), a common condition in some populations (more typically in low/middle income countries), characterised by impaired gut barrier function, intestinal inflammation and microbial translocation [80], is associated with an altered gut microbiome (although the direction of cause and effect is not known). Maternal EED may increase the risk for PTB, LBW, and impaired neurodevelopment [81], although reports are conflicting [82]. Neither gut dysbiosis nor EED have been studied in the context of maternal HBV infection, impact on vertical transmission or impact on children HBEU.

### 7.5. Drug toxicity

Another potential influence on fetal development and outcomes is exposure to nucleos/tide analogue (NA) agents taken during pregnancy, most commonly tenofovir disoproxil fumarate (TDF). The use of TDF in the context of high viraemia during pregnancy is effective in prevention of vertical transmission [83–85], and a wide literature (from both HBV and HIV) shows that benefits exceed risks. Large studies and meta-analyses have provided reassurance by showing no significant increase in congenital malformations or infant bone mineral density (BMD) up to one year of age [85–89]. Tenofovir safety in pregnancy has also been demonstrated in trials of HIV pre-exposure prophylaxis (PrEP), in which there is no evidence of adverse pregnancy outcomes or restricted fetal growth [90,91]. Longer-term studies following fetal TDF exposure reported normal physical growth, BMD and neurodevelopment in children up to 192 weeks in China [92], and a Taiwanese study showed comparable long-term growth, renal function, and bone development up to age 6–7 years [93]. On these grounds, it is unlikely that drug exposure has a contributory influence on any adverse outcomes in children HBEU, while there are substantial benefits in prevention of long-term infection.

## 8. Universal risk factors for negative health outcomes in children HBEU

### 8.1. Maternal co-morbidity

Maternal HBV infection may increase the risk of common pregnancy-related maternal comorbidities associated with adverse neonatal outcomes [94,95], including gestational diabetes mellitus (GDM) [33,96–98], pre-eclampsia [33,99] and intrahepatic cholestasis of pregnancy (ICP) [33,100]. It has been hypothesised that HBV drives GDM via raised tumour necrosis factor alpha and ferritin, leading to increased insulin resistance, and is associated with elevated risks of PTB, low Apgar scores, macrosomia, respiratory distress syndrome, neonatal jaundice and admission to neonatal intensive care [101]. Risk of pre-eclampsia is higher with maternal HBV infection, leading to less blood flow to the fetal-placental unit and reduced fetal growth [33,102]. The risk of ICP is higher in HBV infection [100,103], hypothesised to be driven by an interaction between modified immune cell function, hormonal changes and the shared entry receptor/bile acid transporter Na+-taurocholic acid co-transporting polypeptide (NTCP) [100]. It poses risks to the fetus and has been associated with increased risk of PTB and LBW [104,105].

### 8.2. Psychosocial risk factors

HBV infection has been associated with socioeconomic deprivation in a wide variety of settings (e.g., published data from England, Brazil, Japan and Turkey [106–109]). This association is often associated with low advocacy and education for populations at risk, barriers to care for marginalised groups, trends in migration, and a lack of sustained programmatic investment in clinical and public health interventions. ‘Key populations’ are also at increased risk of HBV exposure and infection and negative health outcomes, (for example, including vulnerable migrants and people who inject drugs), in whom a complex interplay of stigma and discrimination, competing health and social priorities, and systematic barriers to care, can adversely affect access to interventions for HBV prevention, diagnosis and treatment [110,111]. While these factors - and the extent of their influence - clearly vary between individuals and settings, parental HBV infection may be a component of a complex milieu of influences on long-term health outcomes, with children suffering from impacts on physical and psychosocial development, health behaviours, nutrition, education, and social exposure and opportunities, irrespective of their own HBV status [112,113]. PTB and LBW are also associated with socioeconomic status [114,115]. To better understand these influences, studies of HBV infection and exposure that record and adjust for socioeconomic factors are required in different geographic and sociodemographic population settings.

HBV-related mortality in adults carries a risk of orphanhood for their children, which is a risk factor for extreme poverty. This risk has been quantified as 1.9-fold (95% CI 1.1–3.3) increased hazard of all-cause mortality and 13.3-fold (95% CI 3.9–45.5) increased hazard of liver-related mortality compared to uninfected in a US study [116], but varies significantly between populations. The impact of HBV diagnosis on adult mental health has been reported with high rates of depression and anxiety and decreased quality of life scores [117–119], which may adversely influence cognitive development, school completion and childhood mental health in their offspring [120,121]. However maternal mental health has not been well studied, with only one retrospective cohort in China suggesting no correlation of HBV with post-partum depression [122].

HBV infection is associated with stigma and discrimination [123], with diverse influences on maternal and family health, education, employment and travel opportunities, health-seeking behaviour, and access to healthcare services [124–126], both for individuals living with infection and for family members [9]. To date, there has been no consistent approach to measurement of stigma in people living with HBV, but a partnership between the World Hepatitis Alliance and the European Centre for Disease Prevention and Control (ECDC) has piloted a stigma survey in Europe (https://www.worldhepatitisalliance.org/stigma/) which will support expansion of data in this field.

A systematic review in 2022 demonstrated high rates of intimate partner violence in pregnant women living with HIV, associated with poor mental health, reduced antiretroviral adherence, and reduced access to healthcare or health-seeking behaviour [127]. Equivalent data are lacking for HBV, but it is possible that some PLWHB are likewise at higher risk of intimate partner violence with health, economic and social disadvantages for their children.

Healthcare systems in most societies exclude certain groups, for whom services are inadequately designed and out of reach. Key populations include refugees and vulnerable migrants, traveller communities, the LGBTQ+ community, sex workers, incarcerated people, and those experiencing homelessness, mental illness and substance addiction. Furthermore there are substantial health inequities by geographic regions, and in certain populations which influence the prevalence, outcomes, and access to treatment for PLWHB [128,129]. Due to complex overlapping risk factors, HBV infection is enriched in these groups, enhancing inequity through a combination of morbidity and inadequate access to healthcare.

Children born to a subgroup of individuals who have contracted HBV through injecting drug use, are at risk of the direct consequences of drug exposure *in utero*, and indirect consequences of poverty, maternal malnutrition, mental health problems and childhood [130,131]. HBV prevalence in people who inject drugs is highly variable by setting (estimated between 2–18%) [132], so this risk intersects with other geographical and societal factors.

The risk factors discussed here are equivalent in children who are HBV exposed and those who are HBEU, and further work is needed to determine if these groups have comparable outcomes, how they compare to matched children in the same populations who are unexposed and uninfected, and how outcomes vary by population and geography.

### 8.3. Reduced breastfeeding in mothers living with HBV infection

HBV can be detected in breast milk, but the virus has not been proven to be infectious, and transmission has not been confirmed in animal models [133]. Guidelines therefore recommend breastfeeding [134], as any small transmission risk should be mitigated by universal HepB-BD vaccination alongside other prophylactic interventions, and the health advantages of breastfeeding outweigh potential risks [135–137]. However, in practice, there may be concerns among both PLWHB and reluctance among healthcare professionals to advocate consistently for universal breastfeeding which could reduce breastfeeding in individuals living with HBV, potentially associated with adverse impacts on infant health [138–141].

### 8.4. Co-infection

HBV coinfection with other blood-borne viruses (HIV, HCV and/or HDV) may impact on maternal and infant health [142,143]. In CHEU, maternal severity of disease has been shown to increase infant morbidity, and it is possible that the acceleration of maternal liver disease caused by co-infection is a contributor to morbidity in children HBEU. Other infections, some of which are more prevalent in people living with HIV, could also contribute to adverse outcomes [5], e.g., toxoplasma, rubella, cytomegalovirus, herpes simplex virus, zika virus, and syphilis [144].

## 9. Geographic and temporal influences on prevalence and outcomes of children HBEU

Differences in regional HBV prevalence and coverage of initiatives to prevent vertical transmission impact the global prevalence of children HBEU. Differences in prevalence reflect the complex milieu of socioeconomic, demographic, host genetic, environmental and other factors [145,146], which also influence education and awareness, accessibility and affordability of diagnostics, perinatal care, and prophylactic interventions, and long-term clinical care for both mothers and infants. Risk factors for adverse birth outcomes are likely to differ between low prevalence vs. endemic settings. These geographically-distributed influences could also modulate the phenotype of children HBEU between populations.

The global population of children HBEU will change over time. One influence is expanding vaccination coverage, such that fewer mothers will be living with HBV infection, lowering overall peripartum exposure. However, given the large undiagnosed population reservoir of HBV, and incomplete coverage of vaccination programmes, immunisation will take many decades to deliver elimination, and there is still a priority need for focus on peripartum management of HBV. Interestingly, there is a converse influence which increases the proportion of infants who are HBEU: as better access to diagnosis and preventive interventions are implemented peripartum, among all infants born to mothers living with HBV infection, a a smaller proportion will acquire HBV infection vertically and a higher proportion will be HBEU.

## 10. Evidence gaps and limitations

To date, the majority of published evidence addressing the impact of early-life HBV exposure considers maternal and fetal complications during pregnancy, or at delivery, without considering potential repercussions later in infancy and childhood. As much of the global burden of HBV is undiagnosed, many children HBEU are unrecognised, and most of the studies we reviewed did not differentiate between HBEU vs exposed and infected infants. Furthermore, existing studies are heterogeneous and many do not clearly describe or account for other factors that might contribute to PTB, LBW or neurodevelopmental delays. Some adverse outcomes, such as miscarriage or neonatal mortality, preclude determining fetal or infant HBV infection status. On these grounds, we have here presented hypotheses as a narrative format rather than using formal methodology for a systematic review.

Influences and outcomes may be setting-dependent. Studies that inform a better understanding of the immunological environment *in utero*, the influence of maternal microbiome, and factors influencing vaccine response could provide mechanistic insights into diverse outcomes. Such studies are necessary to develop an evidence-base for intervention, but these are currently lacking. Prospective studies are needed to determine whether the immunological changes in children HBEU persist long-term and are associated with altered susceptibility to, and morbidity and mortality from, other infectious diseases.

**Fig 2:**
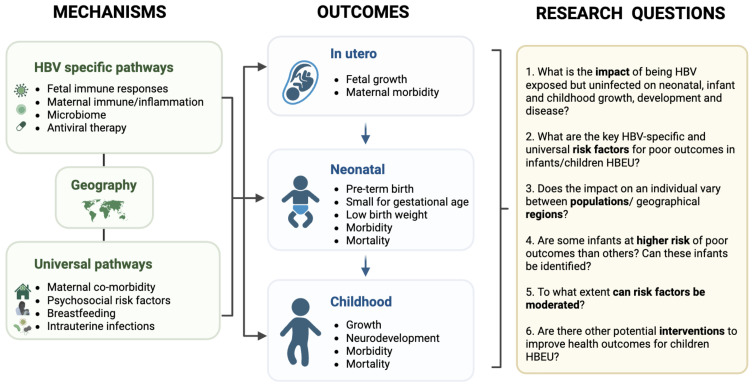
Potential contribution of HBV-specific and universal factors to adverse outcomes *in utero* and long term growth and development and key areas for future research in children HBEU. Created in BioRender. Lumley, S. (2023) https://BioRender.com/m71a556.

There are no data representing children HBEU in the WHO African region for HBV mono-infected individuals, and only one study we could identify of HIV-HBV coinfected individuals [143]. This represents a large data gap in a region with high HBV prevalence, universal risk factors for poor developmental outcomes, high prevalence of exposure to HIV and other pathogens including *Mycobacterium tuberculosis* and malaria [147], and ubiquitous cytomegalovirus infection in infancy, which may collectively contribute to adverse outcomes. Future research should also address regional differences in the outcomes of children HBEU, and seek to assess the impact of maternal health, economic and societal factors. We outline key research gaps for children HBEU in **Fig 2**. Future studies will need to address the challenges of keeping uninfected children under follow-up to assess long term outcomes, with registries held on platforms for data capture, with standardised definitions and time points. As electronic healthcare data collection and links to other datasets advance (e.g., educational outcomes, mental health), there are opportunities for use of routine data to interrogate long-term outcomes. The expansion of data collection has resource implications, but feasibility and efficiency will be improved by linking to efforts to advance towards HBV elimination targets.

## 11. Conclusion and recommendations

This review sought to understand if there are potential health and developmental impacts for children HBEU, and found that evidence is currently lacking. There is a paucity of longitudinal studies to evaluate the long term health outcomes of these children, and the small number of existing studies means that we cannot yet differentiate the health outcomes of children HBEU from infants with HBV infection.

We hypothesise that children HBEU may be at risk of adverse outcomes due to a complex interplay of both HBV-specific and universal mechanisms, with a precedent from the HIV field. Data are needed to establish whether short- and long-term sequelae exist for children HBEU, and to inform evidence-based interventions to mitigate against detrimental outcomes. Establishing a comprehensive understanding of the long-term trajectory of health and well-being among children HBEU throughout childhood into adolescence will require longitudinal observational studies with appropriate control groups to characterise outcomes, identify risk factors and explore underlying mechanistic pathways (**Fig 2**). Interventions during pregnancy and infant vaccination programmes are opportunities for HBV diagnosis, prevention and education, and afford opportunities to enhance maternal and child health [3]. Studies should incorporate areas of different prevalence, represent different environmental influences, explore outcomes in high vs. low/middle income countries, identify high-risk subgroups, and investigate potential mechanistic pathways. Such data are crucial to inform future prevention and intervention studies.

We need evidence to inform guidelines and implementation of public health strategies to support the wellbeing of children HBEU and to mitigate potential long-term health risks, ensuring children HBEU survive and thrive.

## References

[1] Global hepatitis report 2024: action for access in low- and middle-income countries. World Health Organization. 2024. [cited 26 May 2024]. Available from: https://www.who.int/publications/i/item/9789240091672

[2] Global hepatitis report, 2017. World Health Organization. 2017. [cited 30 Sep 2024]. Available: https://www.who.int/publications/i/item/9789241565455

[3] Terrault NA, Levy MT, Cheung KW, Jourdain G. Viral hepatitis and pregnancy. Nat Rev Gastroenterol Hepatol. 2021;18(2):117–30. doi: 10.1038/s41575-020-00361-w 33046891

[4] Wedderburn CJ, Weldon E, Bertran-Cobo C, Rehman AM, Stein DJ, Gibb DM, et al. Early neurodevelopment of HIV-exposed uninfected children in the era of antiretroviral therapy: a systematic review and meta-analysis. Lancet Child Adolesc Health. 2022;6(6):393–408. doi: 10.1016/S2352-4642(22)00071-2 35483380 PMC9090907

[5] Wedderburn CJ, Evans C, Yeung S, Gibb DM, Donald KA, Prendergast AJ. Growth and neurodevelopment of HIV-exposed uninfected children: a conceptual framework. Curr HIV/AIDS Rep. 2019;16:501–13. 31732866 10.1007/s11904-019-00459-0 PMC6920255

[6] Evans C, Jones CE, Prendergast AJ. HIV-exposed, uninfected infants: new global challenges in the era of paediatric HIV elimination. Lancet Infect Dis. 2016;16(6):e92–107. doi: 10.1016/S1473-3099(16)00055-4 27049574

[7] Arikawa S, Rollins N, Newell M-L, Becquet R. Mortality risk and associated factors in HIV-exposed, uninfected children. Trop Med Int Health. 2016;21(6):720–34. doi: 10.1111/tmi.12695 27091659 PMC5021152

[8] Brennan AT, Bonawitz R, Gill CJ, Thea DM, Kleinman M, Useem J, et al. A meta-analysis assessing all-cause mortality in HIV-exposed uninfected compared with HIV-unexposed uninfected infants and children. AIDS. 2016;30(15):2351–60. doi: 10.1097/QAD.0000000000001211 27456985

[9] O’Hara GA, McNaughton AL, Maponga T, Jooste P, Ocama P, Chilengi R, et al. Hepatitis B virus infection as a neglected tropical disease. PLoS Negl Trop Dis. 2017;11(10):e0005842. doi: 10.1371/journal.pntd.0005842 28981505 PMC5628785

[10] Delphin M, Mohammed KS, Downs LO, Lumley SF, Waddilove E, Okanda D, et al. Under-representation of the WHO African region in clinical trials of interventions against hepatitis B virus infection. Lancet Gastroenterol Hepatol. 2024;9(4):383–92. doi: 10.1016/S2468-1253(23)00315-1 38367632 PMC7616036

[11] Hobart C, Pescarini JM, Evans L, Adil HS, Adil ST, Deal A, et al. Hepatitis B infection and immunity in migrant children and pregnant persons in Europe: a systematic review and meta-analysis. J Travel Med. 2024;31(6):taae094. doi: 10.1093/jtm/taae094 38990201 PMC11298050

[12] Introducing a framework for implementing triple elimination of mother-to-child transmission of HIV, syphilis and hepatitis B virus: policy brief. World Health Organization; 2024. [cited 15 Aug 2024]. Available from: https://www.who.int/publications/i/item/9789240086784

[13] Beasley RP, Trepo C, Stevens CE, Szmuness W. The e antigen and vertical transmission of hepatitis B surface antigen. Am J Epidemiol. 1977;105(2):94–8. doi: 10.1093/oxfordjournals.aje.a112370 835566

[14] Wong VC, Ip HM, Reesink HW, Lelie PN, Reerink-Brongers EE, Yeung CY, et al. Prevention of the HBsAg carrier state in newborn infants of mothers who are chronic carriers of HBsAg and HBeAg by administration of hepatitis-B vaccine and hepatitis-B immunoglobulin. Double-blind randomised placebo-controlled study. Lancet. 1984;1(8383):921–6. doi: 10.1016/s0140-6736(84)92388-2 6143868

[15] Okada K, Kamiyama I, Inomata M, Imai M, Miyakawa Y. e antigen and anti-e in the serum of asymptomatic carrier mothers as indicators of positive and negative transmission of hepatitis B virus to their infants. N Engl J Med. 1976;294(14):746–9. doi: 10.1056/NEJM197604012941402 943694

[16] Hepatitis: Preventing mother-to-child transmission of the hepatitis B virus. [cited 26 Jul 2024]. Available from: https://www.who.int/news-room/questions-and-answers/item/hepatitis-preventing-mother-to-child-transmission-of-the-hepatitis-b-virus

[17] Prevention of Mother-to-Child Transmission of Hepatitis B Virus: Guidelines on Antiviral Prophylaxis in Pregnancy. Geneva: World Health Organization; 2023. 32833415

[18] Duri K, Munjoma PT, Mataramvura H, Mazhandu AJ, Chandiwana P, Marere T. Antenatal hepatitis B virus sero-prevalence, risk factors, pregnancy outcomes and vertical transmission rate within 24 months after birth in a high HIV prevalence setting. BMC Infect Dis. 2023;23:736. doi: 10.1186/s12879-023-08523-2 37891471 PMC10612272

[19] Global Vaccine Alliance to deploy six-in-one vaccine to lower-income countries, establish innovative mechanisms to protect against future epidemic threats. 2023 [cited 15 Aug 2024]. Available from: https://www.gavi.org/news/media-room/global-vaccine-alliance-deploy-six-one-vaccine-lower-income-countries

[20] Yang S, Zhong L, Huang L, Lin S, Li Y. Global burden and trends of viral hepatitis among women of childbearing age from 1990 to 2021. Front Microbiol. 2025;16:1553129. 40061861 10.3389/fmicb.2025.1553129 PMC11885505

[21] McNaughton AL, Lourenço J, Bester PA, Mokaya J, Lumley SF, Obolski U, et al. Hepatitis B virus seroepidemiology data for Africa: Modelling intervention strategies based on a systematic review and meta-analysis. PLoS Med. 2020;17(4):e1003068. doi: 10.1371/journal.pmed.1003068 32315297 PMC7173646

[22] Zhao F, Xie X, Tan X, Yu H, Tian M, Lv H. The functions of hepatitis B virus encoding proteins: viral persistence and liver pathogenesis. Front Immunol. 2021;12:691766. doi: 10.3389/fimmu.2021.691766 34456908 PMC8387624

[23] Wang Z, Zhang J, Yang H, Li X, Wen S, Guo Y. Quantitative analysis of HBV DNA level and HBeAg titer in hepatitis B surface antigen positive mothers and their babies: HBeAg passage through the placenta and the rate of decay in babies. J Med Virol. 2003;71:360–6. doi: 10.1002/jmv.10493 12966540

[24] Vranckx R, Alisjahbana A, Meheus A. Hepatitis B virus vaccination and antenatal transmission of HBV markers to neonates. J Viral Hepat. 1999;6(2):135–9. doi: 10.1046/j.1365-2893.1999.00145.x 10607224

[25] Chen T, Wang J, Feng Y, Yan Z, Zhang T, Liu M, et al. Dynamic changes of HBV markers and HBV DNA load in infants born to HBsAg(+) mothers: can positivity of HBsAg or HBV DNA at birth be an indicator for HBV infection of infants? BMC Infect Dis. 2013;13:524. doi: 10.1186/1471-2334-13-524 24195671 PMC3829094

[26] Bernstein SR, Krieger P, Puppala BL, Costello M. Incidence and duration of hepatitis B surface antigenemia after neonatal hepatitis B immunization. J Pediatr. 1994;125(4):621–2. doi: 10.1016/s0022-3476(94)70022-2 7931886

[27] Köksal N, Altinkaya N, Perk Y. Transient hepatitis B surface antigenemia after neonatal hepatitis B immunization. Acta Paediatr. 1996;85(12):1501–2. doi: 10.1111/j.1651-2227.1996.tb13961.x 9001667

[28] Hepatitis B: the green book, chapter 18. In: GOV.UK [Internet]. 2013 [cited 27 Sep 2023]. Available from: https://www.gov.uk/government/publications/hepatitis-b-the-green-book-chapter-18

[29] Mast EE, Margolis HS, Fiore AE, Brink EW, Goldstein ST, Wang SA, et al. A comprehensive immunization strategy to eliminate transmission of hepatitis B virus infection in the United States: recommendations of the Advisory Committee on Immunization Practices (ACIP) part 1: immunization of infants, children, and adolescents. MMWR Recomm Rep. 2005;54(RR-16):1–31. 16371945

[30] Komatsu H, Inui A, Fujisawa T. The role of body fluids in the horizontal transmission of hepatitis B virus via household/close contact. Eur Med J (Chelmsf). 2016:68–75.

[31] Cui A-M, Shao J-G, Li H-B, Shen Y, Chen Z-X, Zhang S, et al. Association of chronic hepatitis B virus infection with preterm birth: our experience and meta-analysis. J Perinat Med. 2017;45(8):933–40. doi: 10.1515/jpm-2016-0201 27875320

[32] Ma X, Sun D, Li C, Ying J, Yan Y. Chronic hepatitis B virus infection and preterm labor(birth) in pregnant women-an updated systematic review and meta-analysis. J Med Virol. 2018;90(1):93–100. doi: 10.1002/jmv.24927 28851115

[33] Afraie M, Moradi G, Zamani K, Azami M, Moradi Y. The effect of hepatitis B virus on the risk of pregnancy outcomes: a systematic review and meta-analysis of cohort studies. Virol J. 2023;20(1):213. doi: 10.1186/s12985-023-02182-0 37710321 PMC10500763

[34] Sirilert S, Traisrisilp K, Sirivatanapa P, Tongsong T. Pregnancy outcomes among chronic carriers of hepatitis B virus. Int J Gynaecol Obstet. 2014;126(2):106–10. doi: 10.1016/j.ijgo.2014.02.019 24834849

[35] Liu J, Zhang S, Liu M, Wang Q, Shen H, Zhang Y. Maternal pre-pregnancy infection with hepatitis B virus and the risk of preterm birth: a population-based cohort study. Lancet Glob Health. 2017;5(6):e624–32. doi: 10.1016/S2214-109X(17)30142-0 28495266

[36] Safety F. Global nutrition targets 2025: low birth weight policy brief. World Health Organization; 2014 [cited 15 Sep 2023]. Available from: https://www.who.int/publications/i/item/WHO-NMH-NHD-14.5

[37] Risnes KR, Vatten LJ, Baker JL, Jameson K, Sovio U, Kajantie E, et al. Birthweight and mortality in adulthood: a systematic review and meta-analysis. Int J Epidemiol. 2011;40(3):647–61. doi: 10.1093/ije/dyq267 21324938

[38] Larroque B, Bertrais S, Czernichow P, Léger J. School difficulties in 20-year-olds who were born small for gestational age at term in a regional cohort study. Pediatrics. 2001;108:111–5. doi: 10.1542/peds.108.1.111 11433062

[39] Zhang XH, Wang Q, Zheng W, Li XH, Jiang QQ, Zhou CF, et al. Early physical growth and disease analysis among children born delivered by HBsAg-positive mothers. Zhonghua Yu Fang Yi Xue Za Zhi. 2017;51(6):496–500. doi: 10.3760/cma.j.issn.0253-9624.2017.06.008 28592092

[40] Ko SC, Schillie SF, Walker T, Veselsky SL, Nelson NP, Lazaroff J. Hepatitis B vaccine response among infants born to hepatitis B surface antigen-positive women. Vaccine. 2014;32:2127–33. doi: 10.1016/j.vaccine.2014.01.099 24560676

[41] Wang J, He Y, Jin D, Liu J, Zheng J, Yuan N, et al. No response to hepatitis B vaccine in infants born to HBsAg(+) mothers is associated to the transplacental transfer of HBsAg. Infect Dis (Lond). 2017;49(8):576–83. doi: 10.1080/23744235.2017.1292541 28276802

[42] Badur S, Lazizi Y, Ugurlu M, Perk Y, Ilter O, Aydinli K, et al. Transplacental passage of hepatitis B virus DNA from hepatitis B e antigen-negative mothers and delayed immune response in newborns. J Infect Dis. 1994;169(3):704–6. doi: 10.1093/infdis/169.3.704 8158060

[43] Tian Y, Kuo C-F, Akbari O, Ou J-HJ. Maternal-derived hepatitis B virus E antigen alters macrophage function in offspring to drive viral persistence after vertical transmission. Immunity. 2016;44(5):1204–14. doi: 10.1016/j.immuni.2016.04.008 27156385 PMC4871724

[44] Milich DR, Chen MK, Hughes JL, Jones JE. The secreted hepatitis B precore antigen can modulate the immune response to the nucleocapsid: a mechanism for persistence. J Immunol. 1998;160(4):2013–21. 9469465

[45] Prendergast AJ, Klenerman P, Goulder PJR. The impact of differential antiviral immunity in children and adults. Nat Rev Immunol. 2012;12(9):636–48. doi: 10.1038/nri3277 22918466

[46] Jiang H, Chen C, Yuan D, Ye X, Chen Y, Han G, et al. The relationship of maternal hepatitis B e antigen and response to vaccination of infants born to women with chronic infection. BMC Pregnancy Childbirth. 2023;23(1):518. doi: 10.1186/s12884-023-05815-y 37454068 PMC10349460

[47] Huang H, Ning M, Liu J, Chen J, Feng J, Dai Y, et al. Comparison of antibody response to hepatitis B vaccination in infants with positive or negative maternal hepatitis B e antigen (HBeAg) in cord blood: implication for the role of HBeAg as an immunotolerogen. Hum Vaccin Immunother. 2019;15(9):2183–6. doi: 10.1080/21645515.2019.1575712 30735449 PMC6773413

[48] Koumbi L, Bertoletti A, Anastasiadou V, Machaira M, Goh W, Papadopoulos NG, et al. Hepatitis B-specific T helper cell responses in uninfected infants born to HBsAg+/HBeAg- mothers. Cell Mol Immunol. 2010;7(6):454–8. doi: 10.1038/cmi.2010.34 20657604 PMC4002954

[49] Hong M, Sandalova E, Low D, Gehring AJ, Fieni S, Amadei B, et al. Trained immunity in newborn infants of HBV-infected mothers. Nat Commun. 2015;6:6588. doi: 10.1038/ncomms7588 25807344 PMC4389241

[50] Benn CS, Fisker AB, Rieckmann A, Sørup S, Aaby P. Vaccinology: time to change the paradigm? Lancet Infect Dis. 2020;20:e274–83. doi: 10.1016/S1473-3099(19)30742-X 32645296

[51] Netea MG, Quintin J, van der Meer JWM. Trained immunity: a memory for innate host defense. Cell Host Microbe. 2011;9(5):355–61. doi: 10.1016/j.chom.2011.04.006 21575907

[52] Barton ES, White DW, Cathelyn JS, Brett-McClellan KA, Engle M, Diamond MS, et al. Herpesvirus latency confers symbiotic protection from bacterial infection. Nature. 2007;447(7142):326–9. doi: 10.1038/nature05762 17507983

[53] Estes ML, McAllister AK. Maternal immune activation: Implications for neuropsychiatric disorders. Science. 2016;353(6301):772–7. doi: 10.1126/science.aag3194 27540164 PMC5650490

[54] Weaver ICG, Cervoni N, Champagne FA, D’Alessio AC, Sharma S, Seckl JR, et al. Epigenetic programming by maternal behavior. Nat Neurosci. 2004;7(8):847–54. doi: 10.1038/nn1276 15220929

[55] Bale TL. Epigenetic and transgenerational reprogramming of brain development. Nat Rev Neurosci. 2015;16:332–44. doi: 10.1038/nrn3818 25921815 PMC7064155

[56] Weber-Stadlbauer U. Epigenetic and transgenerational mechanisms in infection-mediated neurodevelopmental disorders. Transl Psychiatry. 2017;7(5):e1113. doi: 10.1038/tp.2017.78 28463237 PMC5534947

[57] Xu D-Z, Yan Y-P, Choi BCK, Xu J-Q, Men K, Zhang J-X, et al. Risk factors and mechanism of transplacental transmission of hepatitis B virus: a case-control study. J Med Virol. 2002;67(1):20–6. doi: 10.1002/jmv.2187 11920813

[58] Bai G, Wang Y, Zhang L, Tang Y, Fu F. The study on the role of hepatitis B virus X protein and apoptosis in HBV intrauterine infection. Arch Gynecol Obstet. 2012;285(4):943–9. doi: 10.1007/s00404-011-2096-2 21986716

[59] Wang JS, Zhu QR. Infection of the fetus with hepatitis B e antigen via the placenta. Lancet. 2000;355(9208):989. doi: 10.1016/S0140-6736(00)90021-7 10768442

[60] Dauby N, Goetghebuer T, Kollmann TR, Levy J, Marchant A. Uninfected but not unaffected: chronic maternal infections during pregnancy, fetal immunity, and susceptibility to postnatal infections. Lancet Infect Dis. 2012;12(4):330–40. doi: 10.1016/S1473-3099(11)70341-3 22364680

[61] Chang M-L, Liaw Y-F. Hepatitis B flares in chronic hepatitis B: pathogenesis, natural course, and management. J Hepatol. 2014;61(6):1407–17. doi: 10.1016/j.jhep.2014.08.033 25178562

[62] Kushner T, Park C, Masand D, Rosenbluth E, Carroll C, Grace M, et al. Prevalence of elevated alanine aminotransferase (ALT) in pregnancy: a cross-sectional labor and delivery-based assessment. Medicine (Baltimore). 2022;101(40):e30408. doi: 10.1097/MD.0000000000030408 36221350 PMC9542988

[63] Bzowej NH, Tran TT, Li R, Belle SH, Smith CI, Khalili M, et al. Total Alanine Aminotransferase (ALT) flares in pregnant North American women with chronic hepatitis b infection: results from a prospective observational study. Am J Gastroenterol. 2019;114(8):1283–91. doi: 10.14309/ajg.0000000000000221 31082876 PMC7132838

[64] Nguyen G, Garcia RT, Nguyen N, Trinh H, Keeffe EB, Nguyen MH. Clinical course of hepatitis B virus infection during pregnancy. Aliment Pharmacol Ther. 2009;29(7):755–64. doi: 10.1111/j.1365-2036.2009.03932.x 19183158

[65] Lee SM, Park JS, Han YJ, Kim W, Bang SH, Kim BJ, et al. Elevated alanine aminotransferase in early pregnancy and subsequent development of gestational diabetes and preeclampsia. J Korean Med Sci. 2020;35(26):e198. doi: 10.3346/jkms.2020.35.e198 32627436 PMC7338210

[66] Fukuda R, Ishimura N, Nguyen TX, Chowdhury A, Ishihara S, Kohge N. The expression of IL-2, IL-4 and interferon-gamma (IFN-gamma) mRNA using liver biopsies at different phases of acute exacerbation of chronic hepatitis B. Clin Exp Immunol. 1995;100:446–51. doi: 10.1111/j.1365-2249.1995.tb03720.x 7774054 PMC1534464

[67] Coley EJL, Hsiao EY. Malnutrition and the microbiome as modifiers of early neurodevelopment. Trends Neurosci. 2021;44(9):753–64. doi: 10.1016/j.tins.2021.06.004 34303552

[68] Buffington SA, Dooling SW, Sgritta M, Noecker C, Murillo OD, Felice DF. Dissecting the contribution of host genetics and the microbiome in complex behaviors. Cell. 2021;184:1740–1756.e16. doi: 10.1016/j.cell.2021.02.009 33705688 PMC8996745

[69] Chandiwana P, Munjoma PT, Mazhandu AJ, Li J, Baertschi I, Wyss J, et al. Antenatal gut microbiome profiles and effect on pregnancy outcome in HIV infected and HIV uninfected women in a resource limited setting. BMC Microbiol. 2023;23(1):4. doi: 10.1186/s12866-022-02747-z 36604616 PMC9817306

[70] Robertson RC, Edens TJ, Carr L, Mutasa K, Gough EK, Evans C, et al. The gut microbiome and early-life growth in a population with high prevalence of stunting. Nat Commun. 2023;14(1):654. doi: 10.1038/s41467-023-36135-6 36788215 PMC9929340

[71] Yang R, Xu Y, Dai Z, Lin X, Wang H. The Immunologic Role of Gut Microbiota in Patients with Chronic HBV Infection. J Immunol Res. 2018;2018:2361963. doi: 10.1155/2018/2361963 30148173 PMC6083645

[72] Wang K, Zhang Z, Mo ZS, Yang XH, Lin BL, Peng L. Gut microbiota as prognosis markers for patients with HBV-related acute-on-chronic liver failure. Gut Microbes. 2021;13:1–15. 10.1080/19490976.2021.1921925 PMC8143260 34006193

[73] Sandler NG, Koh C, Roque A, Eccleston JL, Siegel RB, Demino M, et al. Host response to translocated microbial products predicts outcomes of patients with HBV or HCV infection. Gastroenterology. 2011;141(4):1220–30, 1230.e1-3. doi: 10.1053/j.gastro.2011.06.063 21726511 PMC3186837

[74] Chou H-H, Chien W-H, Wu L-L, Cheng C-H, Chung C-H, Horng J-H, et al. Age-related immune clearance of hepatitis B virus infection requires the establishment of gut microbiota. Proc Natl Acad Sci U S A. 2015;112(7):2175–80. doi: 10.1073/pnas.1424775112 25646429 PMC4343154

[75] Wang J, Wang Y, Zhang X, Liu J, Zhang Q, Zhao Y, et al. Gut microbial dysbiosis is associated with altered hepatic functions and serum metabolites in chronic hepatitis B Patients. Front Microbiol. 2017;8:2222. doi: 10.3389/fmicb.2017.02222 29180991 PMC5693892

[76] Ouyang J, Zaongo SD, Zhang X, Qi M, Hu A, Wu H. Microbiota-mediated immunity abnormalities facilitate hepatitis B Virus co-infection in people living with HIV: a review. Front Immunol. 2021;12:755890. doi: 10.3389/fimmu.2021.755890 35069530 PMC8770824

[77] Ren Y-D, Ye Z-S, Yang L-Z, Jin L-X, Wei W-J, Deng Y-Y, et al. Fecal microbiota transplantation induces hepatitis B virus e-antigen (HBeAg) clearance in patients with positive HBeAg after long-term antiviral therapy. Hepatology. 2017;65(5):1765–8. doi: 10.1002/hep.29008 28027582

[78] Jašarević E, Bale TL. Prenatal and postnatal contributions of the maternal microbiome on offspring programming. Front Neuroendocrinol. 2019;55:100797. doi: 10.1016/j.yfrne.2019.100797 31574280

[79] Sajdel-Sulkowska EM. The impact of maternal gut microbiota during pregnancy on fetal gut-brain axis development and life-long health outcomes. Microorganisms. 2023;11(9):2199. doi: 10.3390/microorganisms11092199 37764043 PMC10538154

[80] Tickell KD, Atlas HE, Walson JL. Environmental enteric dysfunction: a review of potential mechanisms, consequences and management strategies. BMC Med. 2019;17: 181. doi: 10.1186/s12916-019-1417-3 31760941 PMC6876067

[81] Cowardin CA, Syed S, Iqbal N, Jamil Z, Sadiq K, Iqbal J, et al. Environmental enteric dysfunction: gut and microbiota adaptation in pregnancy and infancy. Nat Rev Gastroenterol Hepatol. 2023;20(4):223–37. doi: 10.1038/s41575-022-00714-7 36526906 PMC10065936

[82] Lauer JM, Kirby MA, Muhihi A, Ulenga N, Aboud S, Liu E, et al. Assessing environmental enteric dysfunction via multiplex assay and its relation to growth and development among HIV-exposed uninfected Tanzanian infants. PLoS Negl Trop Dis. 2023;17(3):e0011181. doi: 10.1371/journal.pntd.0011181 36943785 PMC10030025

[83] Zou H, Chen Y, Duan Z, Zhang H, Pan C. Virologic factors associated with failure to passive-active immunoprophylaxis in infants born to HBsAg-positive mothers. J Viral Hepat. 2012;19(2):e18-25. doi: 10.1111/j.1365-2893.2011.01492.x 22239517

[84] Pan CQ, Duan Z, Dai E, Zhang S, Han G, Wang Y, et al. Tenofovir to Prevent Hepatitis B Transmission in Mothers with High Viral Load. N Engl J Med. 2016;374(24):2324–34. doi: 10.1056/NEJMoa1508660 27305192

[85] Jourdain G, Ngo-Giang-Huong N, Harrison L, Decker L, Khamduang W, Tierney C. Tenofovir versus placebo to prevent perinatal transmission of hepatitis B. N Engl J Med. 2018;378:911–23. doi: 10.1056/NEJMoa1708131 29514030 PMC5895092

[86] Salvadori N, Fan B, Teeyasoontranon W, Ngo-Giang-Huong N, Phanomcheong S, Luvira A, et al. Maternal and infant bone mineral density 1 year after delivery in a randomized, controlled trial of maternal tenofovir disoproxil fumarate to prevent mother-to-child transmission of hepatitis B virus. Clin Infect Dis. 2019;69(1):144–6. doi: 10.1093/cid/ciy982 30924492 PMC6579954

[87] Hyun MH, Lee Y-S, Kim JH, Je JH, Yoo YJ, Yeon JE, et al. Systematic review with meta-analysis: the efficacy and safety of tenofovir to prevent mother-to-child transmission of hepatitis B virus. Aliment Pharmacol Ther. 2017;45(12):1493–505. doi: 10.1111/apt.14068 28436552

[88] Funk AL, Lu Y, Yoshida K, Zhao T, Boucheron P, van Holten J. Efficacy and safety of antiviral prophylaxis during pregnancy to prevent mother-to-child transmission of hepatitis B virus: a systematic review and meta-analysis. Lancet Infect Dis. 2021;21:70–84. doi: 10.1016/S1473-3099(20)30586-7 32805200

[89] Brown RS Jr, McMahon BJ, Lok ASF, Wong JB, Ahmed AT, Mouchli MA, et al. Antiviral therapy in chronic hepatitis B viral infection during pregnancy: a systematic review and meta-analysis. Hepatology. 2016;63(1):319–33. doi: 10.1002/hep.28302 26565396

[90] Heffron R, Mugo N, Hong T, Celum C, Marzinke MA, Ngure K. Pregnancy outcomes and infant growth among babies with in-utero exposure to tenofovir-based preexposure prophylaxis for HIV prevention. AIDS. 2018;32:1707–13. doi: 10.1097/QAD.0000000000001867 30001244 PMC6086376

[91] Moodley D, Lombard C, Govender V, Naidoo M, Desmond AC, Naidoo K, et al. Pregnancy and neonatal safety outcomes of timing of initiation of daily oral tenofovir disoproxil fumarate and emtricitabine pre-exposure prophylaxis for HIV prevention (CAP016): an open-label, randomised, non-inferiority trial. Lancet HIV. 2023;10:e154–63. doi: 10.1016/S2352-3018(22)00369-1 36746169

[92] Pan CQ, Dai E, Duan Z, Han G, Zhao W, Wang Y, et al. Long-term safety of infants from mothers with chronic hepatitis B treated with tenofovir disoproxil in China. Gut. 2022;71(4):798–806. doi: 10.1136/gutjnl-2020-322719 33789963

[93] Wen W-H, Chen H-L, Shih TT-F, Wu J-F, Ni Y-H, Lee C-N, et al. Long-term growth and bone development in children of HBV-infected mothers with and without fetal exposure to tenofovir disoproxil fumarate. J Hepatol. 2020;72: 1082–1087. doi: 10.1016/j.jhep.2020.01.021 32044401

[94] Kumar M, Abbas Z, Azami M, Belopolskaya M, Dokmeci AK, Ghazinyan H, et al. Asian Pacific association for the study of liver (APASL) guidelines: hepatitis B virus in pregnancy. Hepatol Int. 2022;16(2):211–53. doi: 10.1007/s12072-021-10285-5 35113359

[95] Tan J, Liu X, Mao X, Yu J, Chen M, Li Y. HBsAg positivity during pregnancy and adverse maternal outcomes: a retrospective cohort analysis. J Viral Hepat. 2016;23:812–9. doi: 10.1111/jvh.12545 27167604

[96] Tan J, Mao X, Zhang G, Wang W, Pan T, Liu X, et al. Hepatitis B surface antigen positivity during pregnancy and risk of gestational diabetes mellitus: a systematic review and meta-analysis. J Viral Hepat. 2018;25(11):1372–83. doi: 10.1111/jvh.12964 29968379

[97] Chen B, Wang Y, Lange M, Kushner T. Hepatitis C is associated with more adverse pregnancy outcomes than hepatitis B: a 7-year national inpatient sample study. Hepatol Commun. 2022;6(9):2465–73. doi: 10.1002/hep4.2002 35748104 PMC9426407

[98] Peng S, Wan Z, Lin X, Li X, Du Y. Maternal hepatitis B surface antigen carrier status increased the incidence of gestational diabetes mellitus. BMC Infect Dis. 2019;19:147. doi: 10.1186/s12879-019-3749-1 30760217 PMC6373004

[99] Keramat A, Younesian M, Gholami Fesharaki F, Hasani M, Mirzaei S, Ebrahimi E. Inactive hepatitis B carrier and pregnancy outcomes: a systematic review and meta-analysis. Iran J Public Health. 2017;46:468–74. 28540262 PMC5439035

[100] Jiang R, Wang T, Yao Y, Zhou F, Huang X. Hepatitis B infection and intrahepatic cholestasis of pregnancy: a systematic review and meta-analysis. Medicine (Baltimore). 2020;99(31):e21416. doi: 10.1097/MD.0000000000021416 32756142 PMC7402766

[101] Ye W, Luo C, Huang J, Li C, Liu Z, Liu F. Gestational diabetes mellitus and adverse pregnancy outcomes: systematic review and meta-analysis. BMJ. 2022;377:e067946. doi: 10.1136/bmj-2021-067946 35613728 PMC9131781

[102] Dimitriadis E, Rolnik DL, Zhou W, Estrada-Gutierrez G, Koga K, Francisco RPV, et al. Pre-eclampsia. Nat Rev Dis Primers. 2023;9(1):8. doi: 10.1038/s41572-023-00417-6 36797292

[103] Hu Y, Ding Y-L, Yu L. The impact of intrahepatic cholestasis of pregnancy with hepatitis B virus infection on perinatal outcomes. Ther Clin Risk Manag. 2014;10:381–5. doi: 10.2147/TCRM.S61530 24920912 PMC4043812

[104] Wu K, Yin B, Li S, Zhu X, Zhu B. Prevalence, risk factors and adverse perinatal outcomes for Chinese women with intrahepatic cholestasis of pregnancy: a large cross-sectional retrospective study. Ann Med. 2022;54(1):2966–74. doi: 10.1080/07853890.2022.2136400 36271887 PMC9624205

[105] Ovadia C, Seed PT, Sklavounos A, Geenes V, Di Ilio C, Chambers J, et al. Association of adverse perinatal outcomes of intrahepatic cholestasis of pregnancy with biochemical markers: results of aggregate and individual patient data meta-analyses. Lancet. 2019;393:899–909. doi: 10.1016/S0140-6736(18)31877-4 30773280 PMC6396441

[106] Guimarães LC da C, Brunini S, Guimarães RA, Galdino-Júnior H, Minamisava R, da Cunha VE, et al. Epidemiology of hepatitis B virus infection in people living in poverty in the central-west region of Brazil. BMC Public Health. 2019;19(1):443. doi: 10.1186/s12889-019-6828-8 31035990 PMC6489193

[107] Okui T, Nakashima N. Analysis of the association between areal socioeconomic deprivation levels and viral hepatitis B and C infections in Japanese municipalities. BMC Public Health. 2022;22(1):681. doi: 10.1186/s12889-022-13089-w 35392863 PMC8991792

[108] Tosun S, Aygün O, Özdemir HÖ, Korkmaz E, Özdemir D. The impact of economic and social factors on the prevalence of hepatitis B in Turkey. BMC Public Health. 2018;18(1):649. doi: 10.1186/s12889-018-5575-6 29789002 PMC5964685

[109] Campbell C, Wang T, Gillespie I, Barnes E, Matthews PC. Analysis of primary care electronic health record data of people living with hepatitis B virus: infection and hepatocellular carcinoma risk associated with socio-economic deprivation. Public Health. 2024;226:215–27. doi: 10.1016/j.puhe.2023.10.036 38091810 PMC7615551

[110] Martyn E, Eisen S, Longley N, Harris P, Surey J, Norman J, et al. The forgotten people: Hepatitis B virus (HBV) infection as a priority for the inclusion health agenda. Elife. 2023;12:e81070. doi: 10.7554/eLife.81070 36757862 PMC9910830

[111] Im YR, Mohammed KS, Martyn E, Lumley S, Ko J, Mokaya J, et al. Social, clinical and biological barriers to hepatitis B virus suppression with nucleos/tide analogue therapy: who is at risk and what should we do about it?. Sex Transm Infect. 2024;100(5):259–63. doi: 10.1136/sextrans-2023-056089 39059818

[112] Kaplan GA. Social Determinants of Health, 2nd Edition. Marmot M, Wilkinson R, editors. Oxford: Oxford University Press; 2006. pp. 376.

[113] NICE. Childhood disadvantage and adult health: A lifecourse framework Childhood disadvantage and adult health: A lifecourse framework. NICE; [cited 25 Apr 2023]. Available from: https://citeseerx.ist.psu.edu/document?repid=rep1&type=pdf&doi=657b5a13aa979edf298269128383879935d58e2e

[114] Kramer MS, Séguin L, Lydon J, Goulet L. Socio-economic disparities in pregnancy outcome: why do the poor fare so poorly? Paediatr Perinat Epidemiol. 2000;14(3):194–210. doi: 10.1046/j.1365-3016.2000.00266.x 10949211

[115] Weightman AL, Morgan HE, Shepherd MA, Kitcher H, Roberts C, Dunstan FD. Social inequality and infant health in the UK: systematic review and meta-analyses. BMJ Open. 2012;2(3):e000964. doi: 10.1136/bmjopen-2012-000964 22700833 PMC3378945

[116] Zhou K, Dodge JL, Grab J, Poltavskiy E, Terrault NA. Mortality in adults with chronic hepatitis B infection in the United States: a population-based study. Aliment Pharmacol Ther. 2020;52(2):382–9. doi: 10.1111/apt.15803 32432816 PMC7935406

[117] Alian S, Masoudzadeh A, Khoddad T, Dadashian A, Ali Mohammadpour R. Depression in hepatitis B and C, and its correlation with hepatitis drugs consumption (interfron/lamivodin/ribaverin). Iran J Psychiatry Behav Sci. 2013;7(1):24–9. 24644496 PMC3939977

[118] Atesci FC, Cetin BC, Oguzhanoglu NK, Karadag F, Turgut H. Psychiatric disorders and functioning in hepatitis B virus carriers. Psychosomatics. 2005;46(2):142–7. doi: 10.1176/appi.psy.46.2.142 15774953

[119] Altindag A, Cadirci D, Sirmatel F. Depression and health related quality of life in non-cirrhotic chronic hepatitis B patients and hepatitis B carriers. Neurosciences (Riyadh). 2009;14(1):56–9. 21048575

[120] Sherr L, Cluver LD, Betancourt TS, Kellerman SE, Richter LM, Desmond C. Evidence of impact: health, psychological and social effects of adult HIV on children. AIDS. 2014;28 Suppl 3:S251–9. doi: 10.1097/QAD.0000000000000327 24991898

[121] Desmond C, Bruce F, Tomlinson M, Marlow MB, Aber JL, Ouifki R, et al. Modelling the long-term impacts on affected children of adult HIV: benefits, challenges and a possible approach. AIDS. 2014;28 Suppl 3:S269–75. doi: 10.1097/QAD.0000000000000329 24991900

[122] Huang W, Wu X, Yao Z, Gu Y, Lai X, Meng L, et al. Investigating the relationship between hepatitis B virus infection and postpartum depression in Chinese women: a retrospective cohort study. Front Public Health. 2023;11:1214151. doi: 10.3389/fpubh.2023.1214151 38094232 PMC10716447

[123] Mokaya J, McNaughton AL, Burbridge L, Maponga T, O’Hara G, Andersson M, et al. A blind spot? Confronting the stigma of hepatitis B virus (HBV) infection - A systematic review. Wellcome Open Res. 2018;3:29. doi: 10.12688/wellcomeopenres.14273.2 30483598 PMC6234740

[124] Tu T, Block JM, Wang S, Cohen C, Douglas MW. The lived experience of chronic hepatitis B: a broader view of its impacts and why we need a cure. Viruses. 2020;12. doi: 10.3390/v12050515 PMC7290920 32392763

[125] Freeland C, Farrell S, Kumar P, Kamischke M, Jackson M, Bodor S, et al. Common concerns, barriers to care, and the lived experience of individuals with hepatitis B: a qualitative study. BMC Public Health. 2021;21(1):1004. doi: 10.1186/s12889-021-11093-0 34044808 PMC8161662

[126] Adjei CA, Stutterheim SE, Naab F, Ruiter RAC. “To die is better than to tell”: reasons for and against disclosure of chronic hepatitis B status in Ghana. BMC Public Health. 2020;20(1):663. doi: 10.1186/s12889-020-08811-5 32398150 PMC7216649

[127] Yonga AM, Kiss L, Onarheim KH. A systematic review of the effects of intimate partner violence on HIV-positive pregnant women in sub-Saharan Africa. BMC Public Health. 2022;22:220. doi: 10.1186/s12889-022-12619-w 35114964 PMC8815228

[128] Khalili M, Leonard KR, Ghany MG, Hassan M, Roberts LR, Sterling RK, et al. Racial Disparities in Treatment Initiation and Outcomes of Chronic Hepatitis B Virus Infection in North America. JAMA Netw Open. 2023;6(4):e237018. doi: 10.1001/jamanetworkopen.2023.7018 37036707 PMC10087055

[129] Ward JW, Byrd KK. Hepatitis B in the United States: a major health disparity affecting many foreign-born populations. Hepatology. 2012;56(2):419–21. doi: 10.1002/hep.25799 22532028

[130] Guille C, Aujla R. Developmental consequences of prenatal substance use in children and adolescents. J Child Adolesc Psychopharmacol. 2019;29:479–86. doi: 10.1089/cap.2018.0177 31038354

[131] Popova S, Charness ME, Burd L, Crawford A, Hoyme HE, Mukherjee RAS, et al. Fetal alcohol spectrum disorders. Nat Rev Dis Primers. 2023;9(1):11. doi: 10.1038/s41572-023-00420-x 36823161

[132] Syangbo A, Hickman M, Colledge-Frisby S, Leung J, Grebely J, Larney S, et al. Associations between the prevalence of chronic hepatitis B among people who inject drugs and country-level characteristics: An ecological analysis. Drug Alcohol Rev. 2023;42(3):569–81. doi: 10.1111/dar.13595 36600489 PMC10728688

[133] Van de Perre P, Molès J-P, Nagot N, Tuaillon E, Ceccaldi P-E, Goga A, et al. Revisiting Koch’s postulate to determine the plausibility of viral transmission by human milk. Pediatr Allergy Immunol. 2021;32(5):835–42. doi: 10.1111/pai.13473 33594740 PMC8359252

[134] Chen X, Chen J, Wen J, Xu C, Zhang S, Zhou Y-H, et al. Breastfeeding is not a risk factor for mother-to-child transmission of hepatitis B virus. PLoS One. 2013;8(1):e55303. doi: 10.1371/journal.pone.0055303 23383145 PMC3557270

[135] Kramer MS, Kakuma R. Optimal duration of exclusive breastfeeding. Cochrane Database Syst Rev. 2012;2012:CD003517. doi: 10.1002/14651858.CD003517.pub2 11869667

[136] Effect of breastfeeding on infant and child mortality due to infectious diseases in less developed countries: a pooled analysis. WHO Collaborative Study Team on the Role of Breastfeeding on the Prevention of Infant Mortality. Lancet. 2000;355(9202):451–5. 10841125

[137] Ip S, Chung M, Raman G, Chew P, Magula N, DeVine D. Breastfeeding and maternal and infant health outcomes in developed countries. Evid Rep Technol Assess. 2007:1–186. 17764214 PMC4781366

[138] Petrova M, Kamburov V. Breastfeeding and chronic HBV infection: clinical and social implications. World J Gastroenterol. 2010;16:5042–6. doi: 10.3748/wjg.v16.i40.5042 20976840 PMC2965280

[139] Chen S, Li L, Sun Q, Chen S, Cheng J, Xiong S. Effect of IMB model combined with spousal support breastfeeding intervention on PBSES score and breastfeeding rate of primipara with chronic hepatitis B virus infection. Biomed Res Int. 2022;2022:9661408. doi: 10.1155/2022/9661408 36158886 PMC9499791

[140] Li E-M, Xiao L-X, Xu Z, Mo Z-S, Li J-Q, Mei Y-Y, et al. Factors associated with non-compliance with breastfeeding recommendation: a retrospective survey in hepatitis B virus-infected mothers who had taken Nucleos(t)ide analogs during pregnancy. BMC Pregnancy Childbirth. 2021;21(1):551. doi: 10.1186/s12884-021-04020-z 34384374 PMC8359301

[141] Tong Leung VK, Lao TT, Suen SSH, Chan OK, Singh Sahota D, Lau TK, et al. Breastfeeding initiation: is this influenced by maternal hepatitis B infection? J Matern Fetal Neonatal Med. 2012;25(11):2390–4. doi: 10.3109/14767058.2012.697941 22694367

[142] Li C, Yang Y, Wang Y, Dong S, Yang Y, Shi Y, et al. Impact of maternal HIV-HBV coinfection on pregnancy outcomes in an underdeveloped rural area of southwest China. Sex Transm Infect. 2020;96(7):509–15. doi: 10.1136/sextrans-2019-054295 31911426

[143] Bhattacharya D, Guo R, Tseng C-H, Emel L, Sun R, Chiu S-H, et al. Maternal HBV viremia and association with adverse infant outcomes in women living with HIV and HBV. Pediatr Infect Dis J. 2021;40(2):e56–61. doi: 10.1097/INF.0000000000002980 33181788 PMC7855346

[144] Wu S, Wang J, Guo Q, Lan H, Sun Y, Ren M, et al. Prevalence of human immunodeficiency virus, syphilis, and hepatitis B and C virus infections in pregnant women: a systematic review and meta-analysis. Clin Microbiol Infect. 2023;29(8):1000–7. doi: 10.1016/j.cmi.2023.03.002 36921717

[145] Wilson ME. Geography of Infectious Diseases. In: Cohen J, Powderly WG, Opal SM, editors. Infectious Diseases. 4th ed. Elsevier; 2017. pp. 938–947.e1.

[146] Veronese P, Dodi I, Esposito S, Indolfi G. Prevention of vertical transmission of hepatitis B virus infection. World J Gastroenterol. 2021;27(26):4182–93. doi: 10.3748/wjg.v27.i26.4182 34326618 PMC8311536

[147] Adland E, Klenerman P, Goulder P, Matthews PC. Ongoing burden of disease and mortality from HIV/CMV coinfection in Africa in the antiretroviral therapy era. Front Microbiol. 2015;6:1016. doi: 10.3389/fmicb.2015.01016 26441939 PMC4585099

[148] Huang S, Wang J, Xiong Y, Liu C, Qi Y, Zou K. Impact of maternal hepatitis B carrier status on congenital abnormalities: a systematic review and meta-analysis. BMJ Open. 2023;13:e066017. doi: 10.1136/bmjopen-2022-066017 PMC10069551 36977541

[149] Shrivastava S, TrehanPati N, Patra S, Kottilil S, Pande C, Trivedi SS, et al. Increased regulatory T cells and impaired functions of circulating CD8 T lymphocytes is associated with viral persistence in Hepatitis B virus-positive newborns. J Viral Hepat. 2013;20(8):582–91. doi: 10.1111/jvh.12078 23808997

[150] Global health sector strategies on HIV, viral hepatitis and sexually transmitted infections for the period 2022-2030. [cited 30 Sep 2024]. Available from: https://www.who.int/publications/i/item/9789240053779

[151] Guidelines for the prevention, diagnosis, care and treatment for people with chronic hepatitis B infection. World Health Organization; 2024. [cited 30 Sep 2024]. Available from: https://www.who.int/publications/i/item/9789240090903 40424433

[152] Consolidated guidelines on HIV prevention, testing, treatment, service delivery and monitoring: recommendations for a public health approach. World Health Organization; 2021 [cited 30 Sep 2024]. Available from: https://www.who.int/publications/i/item/9789240031593 34370423

[153] Cardenas MC, Farnan S, Hamel BL, Mejia Plazas MC, Sintim-Aboagye E, Littlefield DR, et al. Prevention of the vertical transmission of HIV; a recap of the journey so far. Viruses. 2023;15(4):849. doi: 10.3390/v15040849 37112830 PMC10142818

[154] Terrault NA, Cheung KW, Levy MT, Jourdain G. Reply to “Evidence against in utero transmission of hepatitis B virus”. Nat Rev Gastroenterol Hepatol. 2021;18(6):445–6. doi: 10.1038/s41575-021-00456-y 33888888

[155] Zhou Y-H. Evidence against in utero transmission of hepatitis B virus. Nat Rev Gastroenterol Hepatol. 2021;18(6):445. doi: 10.1038/s41575-021-00455-z 33888887

[156] Lee AK, Ip HM, Wong VC. Mechanisms of maternal-fetal transmission of hepatitis B virus. J Infect Dis. 1978;138(5):668–71. doi: 10.1093/infdis/138.5.668 712120

[157] HIV diagnosis and ARV use in HIV-exposed infants: a programmatic update. World Health Organization; 2018 [cited 1 Feb 2024]. Available from: https://www.who.int/publications/i/item/WHO-CDS-HIV-18.17

[158] Hepatitis B vaccination coverage. In: Immunization Data [Internet]. [cited 14 May 2025]. Available from: https://immunizationdata.who.int/global/wiise-detail-page/hepatitis-b-vaccination-coverage

[159] AIDSinfo. [cited 16 May 2025]. Available from: https://aidsinfo.unaids.org/

[160] Ciaranello AL, Park J-E, Ramirez-Avila L, Freedberg KA, Walensky RP, Leroy V. Early infant HIV-1 diagnosis programs in resource-limited settings: opportunities for improved outcomes and more cost-effective interventions. BMC Med. 2011;9:59. doi: 10.1186/1741-7015-9-59 21599888 PMC3129310

